# Construction and evaluation of a finger motor feedback system to improve finger dexterity

**DOI:** 10.3389/fnrgo.2025.1502492

**Published:** 2025-02-26

**Authors:** Shingo Takahashi, Noriko Sakurai, Yuki Kuroiwa, Daishi Takahashi, Naoki Kodama

**Affiliations:** ^1^Department of Healthcare Informatics, Faculty of Health and Welfare, Takasaki University of Health and Welfare, Gunma, Japan; ^2^Department of Radiological Technology, Faculty of Medical Technology, Niigata University of Health and Welfare, Niigata, Japan

**Keywords:** finger tapping, feedback, dexterity, cognitive function, hand training method

## Abstract

**Introduction:**

Recently, a link has been established between cognitive function and hand dexterity in older adults. Declines in cognitive function have been shown to impair performance in finger tapping movements. Research suggest that hand training can improve dexterity, executive function, and cognitive function over time. This underscores the need for effective methods to improve hand and finger dexterity.

**Method:**

In this study, we introduced a new hand training system that provides real-time feedback on finger movements during tapping tasks. We examined the system's impact on the finger dexterity of 32 healthy young participants by using a magnetic sensor finger tapping device (UB-2). During the finger tapping task, the participants performed opening and closing movements either in-phase or anti-phase on both left and right hands for 15 s. They were instructed to tap as quickly as possible. The number of taps, left–right balance, and other relevant data were measured using the UB-2 device.

**Results:**

In terms of the number of tapping, a significant difference was found between 64.4 without feedback and 68.1 with feedback for the simultaneous opening and closing movements in the dominant hand. In the alternating open-close movement, the significant difference was 50.3 without feedback and 53.4 with feedback. The results showed that the system significantly improved the number and frequency of taps for both hands.

**Conclusion:**

The improved tapping performance with feedback suggests that this system can improve hand dexterity.

## 1 Introduction

According to the World Health Organization, ~55 million people worldwide were projected to have dementia by 2019, and this number is expected to rise to 139 million by 2050 (Alzheimer's Disease International, 2023). A relationship has been found between cognitive function and hand dexterity in older adults, revealing that performance in finger tapping movements declines as cognitive function diminishes (Suzumura et al., 2016, 2021). Additionally, finger tapping performance has proven useful in assessing the risk of mild cognitive impairment (MCI) (Suzumura et al., 2022). Training hand dexterity not only improves dexterity and executive function but may also have long-term benefits for cognitive function (Seol et al., 2023). These findings indicate that developing effective methods to improve hand dexterity is crucial for preventing cognitive decline and dementia in older adults.

Biofeedback, neurofeedback, and visual feedback are methods aimed at improving motor function. Biofeedback involves self-regulation through the visual and auditory presentation of physiological responses, utilizing metrics such as heart rate and electromyography (Frank et al., 2010). By contrast, neurofeedback uses electroencephalography data to provide feedback on brain activity (Marzbani et al., 2016). Visual feedback has demonstrated effectiveness in motor learning (Yamamoto et al., 2019) and is widely used in rehabilitation for stroke patients, with numerous studies supporting its efficacy (Zhu et al., 2020; Lee et al., 2013). Notably, visual feedback has been shown to improve tapping frequency (Barallon et al., 2022). Although some studies focused on visual feedback of hand movements (Saunders and Knill, 2004), studies on simple hand movement feedback to improve cognitive function are lacking. Various methods of visual feedback are available, including video-based feedback on movement (Mödinger et al., 2022), force control adjustment as bars (Archer et al., 2018), and line graphs representing gait information (Castro-Medina, 2023). Feedback methods that provide immediate information about movements are called simultaneous visual feedback, and their effectiveness has been previously reported (Yamamoto et al., 2022).

In this study, we developed a system that provides feedback on finger movements during finger tapping tasks as a novel training method to improve hand dexterity. The purpose of this study is to develop a hand movement feedback system and examine whether the system can effectively improve dexterity. The contributions of this paper are as follows:

- Construction of a simple hand-movement feedback system;
- Implementation of hand movement feedback; and
- Validation of hand dexterity in this method.

The rest of the paper is organized as follows. Section 2 describes related studies, Section 3 describes the constructed feedback system and the validation method, Section 4 discusses the validation results, and Section 5 discusses the discussion and limitations of this paper.

## 2 Related work

A relationship reportedly exists between hand and cognitive functions, and devices have been developed to quantitatively evaluate hand function. Suzumura et al. have investigated the relationship between hand and cognitive functions in patients with dementia and MCI using the UB-1, which can quantitatively measure tapping movements using a porcelain sensor (Suzumura et al., 2016). Chen et al. (2023) have developed a system that can quantitatively evaluate hand movements using image recognition for patients with dementia. The purpose of this paper is different, as the system that can be quantitatively evaluated is used to provide feedback to improve hand function.

One method of improving function is visual feedback. Various methods of visual feedback help improve gait and postural control (Levin et al., 2017; Mani et al., 2022). This technique has also been tested in older adults. Yamamoto et al. (2022) tested simultaneous visual feedback in young and older patients in a learning task using grip strength coordination ability and reported that simultaneous visual feedback is also useful for older patients. Furthermore, visual feedback has shown effectiveness in improving hand function (Kim et al., 2021), and performance could be improved by providing hand feedback (Wang et al., 2023). Although various feedback methods have been used this way, we focus on hand movements, which are related to cognitive function and can be used to implement feedback in a simplified manner.

## 3 Materials and methods

### 3.1 Participants and methods

In this study, we developed a hand movement visual feedback system using a magnetic sensor finger tapping device (UB-2, Maxell, Ltd., Tokyo, Japan) and a programming language (Python) to present real-time visual feedback during tapping tasks. Additionally, we evaluated hand dexterity during the implementation of this system. The participants included 32 healthy young adults (16 males and 16 females, mean age 20.6 ± 1.6 years, hand dominant: 29 right, 3 left) who are currently enrolled in college. Exclusion criteria included higher brain dysfunction, motor impairment, or impaired ability to move fingers, but none of the participants fell into these categories. Although 34 measurements were initially taken, two were excluded because of sensor-mounting issues. For our sample size, the effect size between groups was calculated as 0.5, with a statistical significance level (α) of 5% (two-sided) and a statistical power (1-β) of 80%. The final sample size was 34 participants. We compared hand dexterity with and without feedback using the same device. Ethically, all participants were informed about the study's purpose and provided written consent. This study was approved by the Ethical Review Committee of Takasaki University of Health and Welfare.

### 3.2 Finger motor feedback system

**Figure 1:**
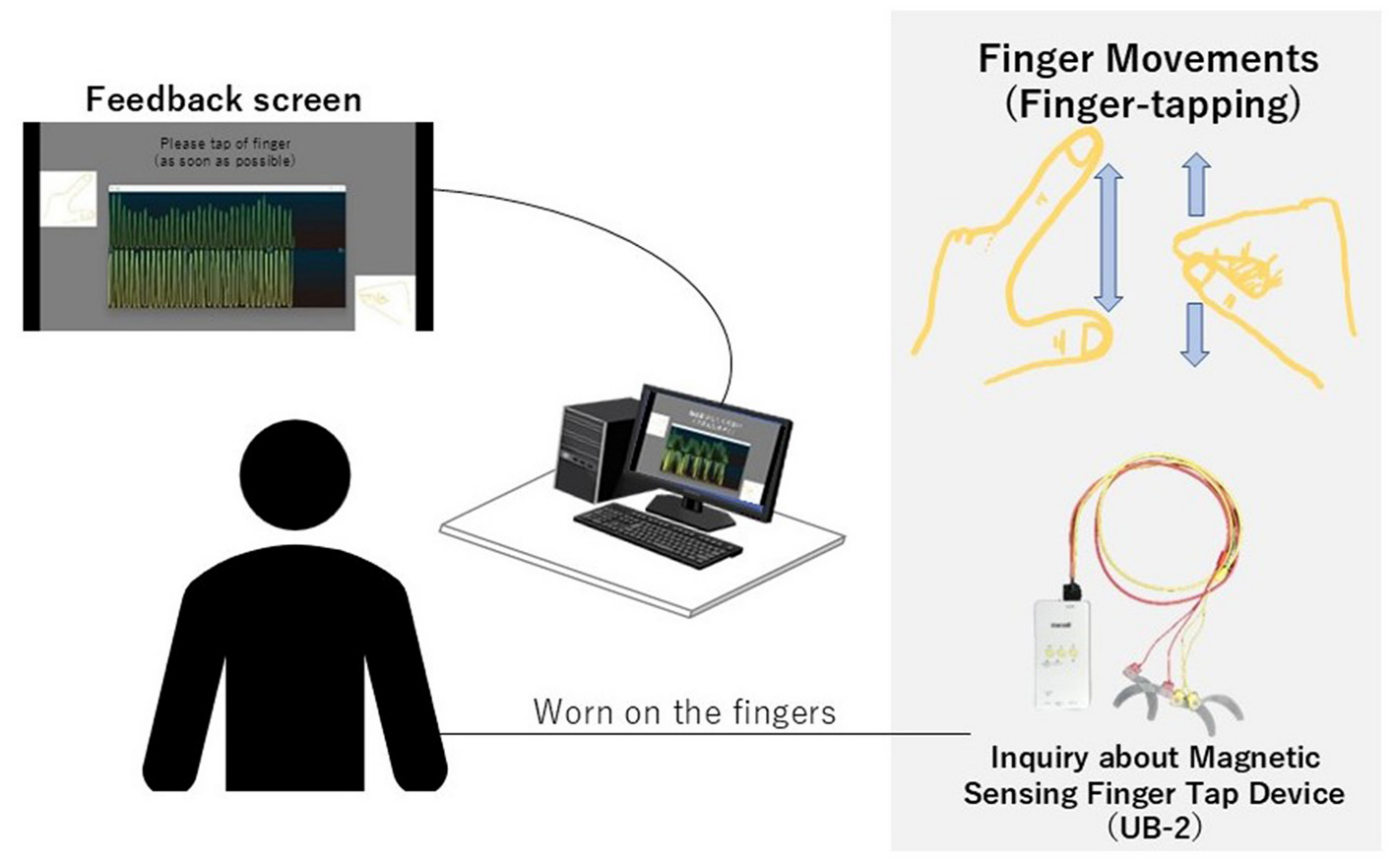
This system enables real-time visualization of hand movements during tapping tasks. The presenting monitor was positioned so that participants could easily view the feedback screen. The waveform display is generated from UB-2 using Python and presented on the screen. Simultaneously, UB-2 records data while tapping task is performed.

**Figure 2:**
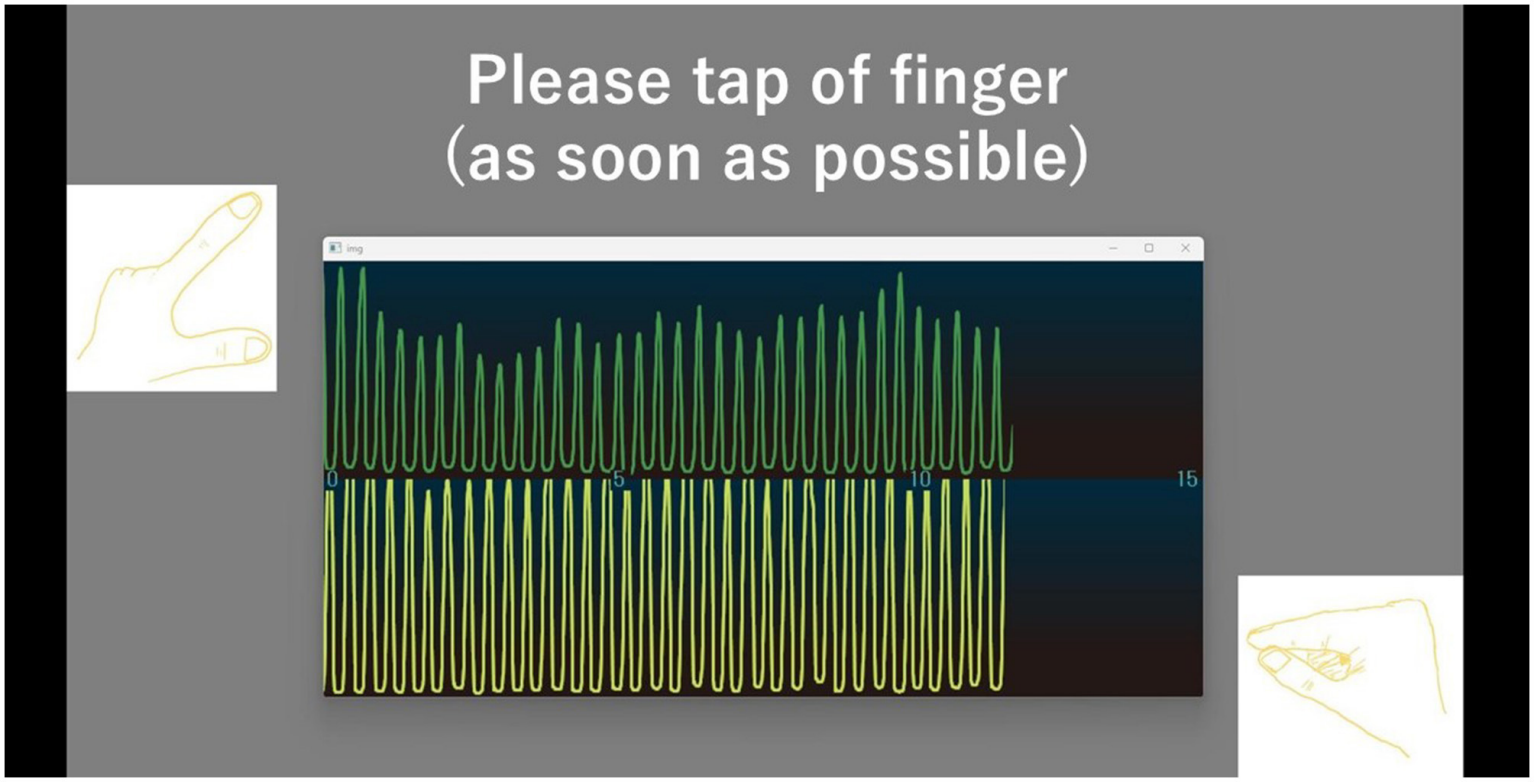
The screen provides feedback on hand movement by displaying waveforms for both the left and right hands. The upper waveform shows the right-hand movement, and the lower waveform shows the left-hand movement. The horizontal axis represents time (second), while the vertical axis indicates finger opening width (cm). When the fingers are closed, the waveform remains horizontal, it rises as the fingers open.

**Figure 3:**
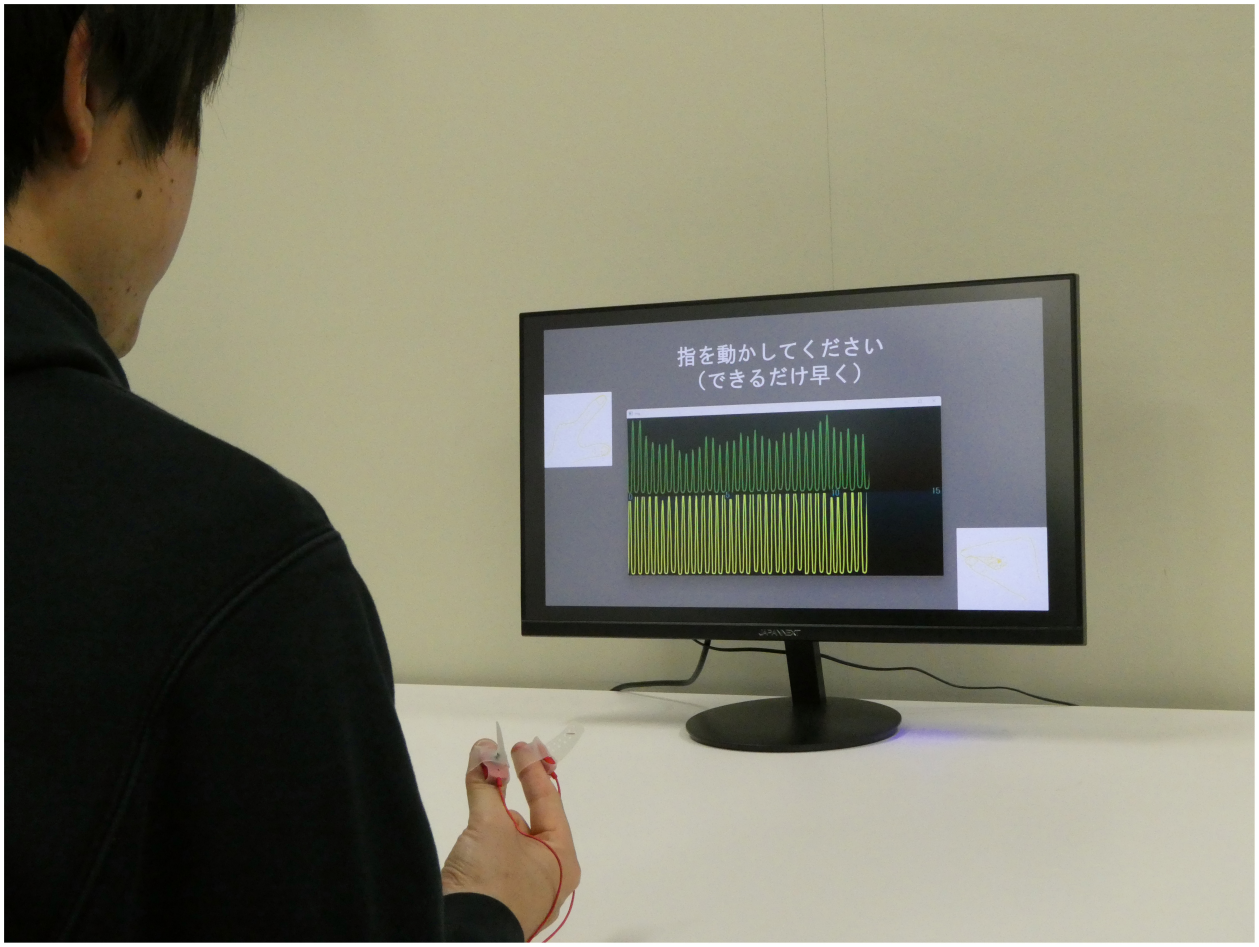
Actual experiment. A monitor is placed in front of the subject, and the information to be feedback is shown on the monitor.

We developed a hand movement feedback system using the magnetic sensor finger tapping device (UB-2). This system visualizes hand movements during tapping tasks through a Python-based program. It comprises the UB-2 device, a computer for measuring and displaying hand motions, and a monitor for real-time feedback. A schematic of the system is shown in Figure 1. The participants received real-time feedback via a segment of the UB-2 measurement screen, where hand movements are represented by waveforms. An example of this feedback display is shown in Figure 2, and the actual experimental scenes are shown in Figure 3.

### 3.3 Finger tapping task

The UB-2 magnetic sensor finger tapping device has sensors attached to the left and right thumbs and index fingers, enabling quantitative evaluation of finger tapping movements. It includes a red and a yellow cable with a porcelain sensor. The red cable is attached to the first and second fingers of the right hand, and the yellow cable is attached to the first and second fingers of the left hand. The device attaches a porcelain sensor to a finger and converts changes in magnetic force from finger tapping into an electrical signal, which is converted into the distance between two fingers. Various parameters are measured from the measured data, including tapping frequency, distance traveled, speed, acceleration, tapping interval, and phase difference (left–right adjustment). During the finger tapping task, the participants performed opening and closing movements either (1) in-phase (simultaneously) or (2) anti-phase (alternately) on both left and right hands for 15 s (Suzumura et al., 2021; Takahashi et al., 2024). “In-phase” is an action in which the same opening and closing movements are performed on the left and right hands. “Anti-phase” is an alternation of different opening and closing movements on the left and right hands (e.g., if one hand closes its fingers, the other hand closes its fingers; if one hand closes its fingers, the other hand closes its fingers). Previous studies that used UB-2 set the finger tapping width at 3–4 cm (Sugioka et al., 2022). Thus, the finger tapping width was set at ~4 cm in the present experiment, and the participants were instructed to tap as quickly as possible. During feedback introduction, the participants were instructed to look only at the monitor without glancing at their hands, so that their own hands were below the monitor. Prior to measurement, the participants were briefed on the tapping behavior, feedback content, and feedback screen, and the experiment was initiated when the participants had fully understood the process. Each tapping motion was practiced for 15 s to ensure proper technique before measurement began. To eliminate the effects of order and habituation, each participant underwent measurements in a different order with and without hand movement feedback. For the measurement setup, the participants were seated comfortably with their forearms resting on a desk from the elbow joint onward, while their third to fifth fingers were gently folded inward.

### 3.4 Data analysis

The data used for the analysis were the mean and standard deviation values for the distance traveled by the hand, the number of taps, and the tap interval, as calculated from the UB-2 instrument. Means and standard deviations were calculated with and without hand movement feedback for each measure. Differences in means with and without balance feedback were compared using t-tests. Statistical analysis was performed using SPSS software (Version 27.0 for Windows; IBM Corp.), with the significance level set to *p* < 0.05.

## 4 Results

The results of alternating and simultaneous tapping for the non-dominant hand, with and without hand motor feedback, are shown in Table 1. The tapping frequency and number were higher during simultaneous tapping than during alternating tapping. Table 2 summarizes the results for the dominant hand, which similarly showed higher values for simultaneous tapping versus alternating tapping. Results related to left–right balance are shown in Table 3. The left–right balance did not significantly differ between simultaneous and alternating tapping.

**Table 1:** Performance of non-dominant hand finger tapping under various conditions: with and without FB, as well as simultaneous and alternating tapping.

	**Ave. of local max. distance [mm]**	**CV of local max. distance [–]**	**Ave. of intervals [sec]**	**CV of inter-tapping interval [–]**
Anti-phase task	65.9 ± 20.1 (58.6–73.1)	0.15 ± 0.06 (0.13–0.18)	0.31 ± 0.05 (0.29–0.32)	0.15 ± 0.07 (0.13–0.18)
Anti-phase task-FB	60.4 ± 21.0 (52.9–68.0)	0.17 ± 0.08 (0.15–0.20)	0.29 ± 0.05 (0.27–0.31)	0.14 ± 0.06 (0.12–0.16)
In-phase task	59.7 ± 18.1 (53.1–66.2)	0.17 ± 0.05 (0.15–0.19)	0.24 ± 0.04 (0.23–0.25)	0.14 ± 0.06 (0.12–0.17)
In-phase task-FB	53.1 ± 17.8 (46.7–59.5)	0.18 ± 0.06 (0.16–0.20)	0.22 ± 0.03 (0.21–0.24)	0.13 ± 0.05 (0.11–0.15)

FB, feedback; Average, average; SD, standard deviation; CV, coefficient of variation.

“Ave. of local max. distance” indicates the amplitude, the larger the value, the better. “CV of local max. distance” is the normalized mean value of the variation of the amplitude; the smaller the value, the better. “Ave. of intervals” is the tapping period, the smaller the value the better. “CV of inter-tapping interval” is the variation of the rhythm; the smaller the value, the better.

**Table 2:** Performance of dominant hand finger tapping under various conditions: with and without FB, as well as simultaneous and alternating tapping.

	**Ave. of local max. distance [mm]**	**CV of local max. distance [–]**	**Ave. of intervals [sec]**	**CV of inter-tapping interval [–]**
Anti-phase task	67.2 ± 24.1 (58.5–75.9)	0.13 ± 0.04 (0.11–0.14)	0.30 ± 0.05 (0.28–0.32)	0.12 ± 0.05 (0.10–0.14)
Anti-phase task-FB	63.3 ± 26.4 (53.7–72.8)	0.13 ± 0.05 (0.12–0.15)	0.28 ± 0.05 (0.27–0.30)	0.11 ± 0.04 (0.10–0.13)
In-phase task	59.4 ± 20.9 (51.9–66.9)	0.17 ± 0.10 (0.13–0.20)	0.24 ± 0.04 (0.22–0.25)	0.12 ± 0.08 (0.09–0.15)
In-phase task-FB	52.3 ± 19.8 (45.1–59.4)	0.14 ± 0.05 (0.12–0.16)	0.22 ± 0.03 (0.21–0.23)	0.11 ± 0.04 (0.09–0.12)

FB, feedback; Average, average; SD, standard deviation; CV, coefficient of variation.

**Table 3:** Results related to left–right balance.

	**Avg. of phase difference between the left hand and right hand tapping**	**Standard deviation of phase difference between the left hand and right hand tapping**
Anti-phase task	179.9 ± 21.2 (172.3–187.5)	33.2 ± 13.0 (28.5–37.9)
Anti-phase task-FB	178.1 ± 22.8 (169.9–186.4)	32.8 ± 11.2 (28.8–36.8)
In-phase task	−24.2 ± 27.0 (−33.9 to −14.5)	48.1 ± 32.4 (36.4–59.7)
In-phase task-FB	−22.9 ± 24.6 (−31.7 to −14.0)	38.8 ± 12.8 (34.2–43.4)

“Avg. of phase difference between the left hand and right hand tapping” is a measure of bilateral misalignment, which should be 0 degrees for simultaneous tasks and 180 degrees for bilateral alternating tasks. It should be 0 degrees for the simultaneous task and 180 degrees for the bimanual task. The “Standard deviation of phase difference between the left hand and right hand tapping” is a measure of the variability of phase difference between the left hand and right hand tapping; the smaller the value, the better.

**Figure 4:**
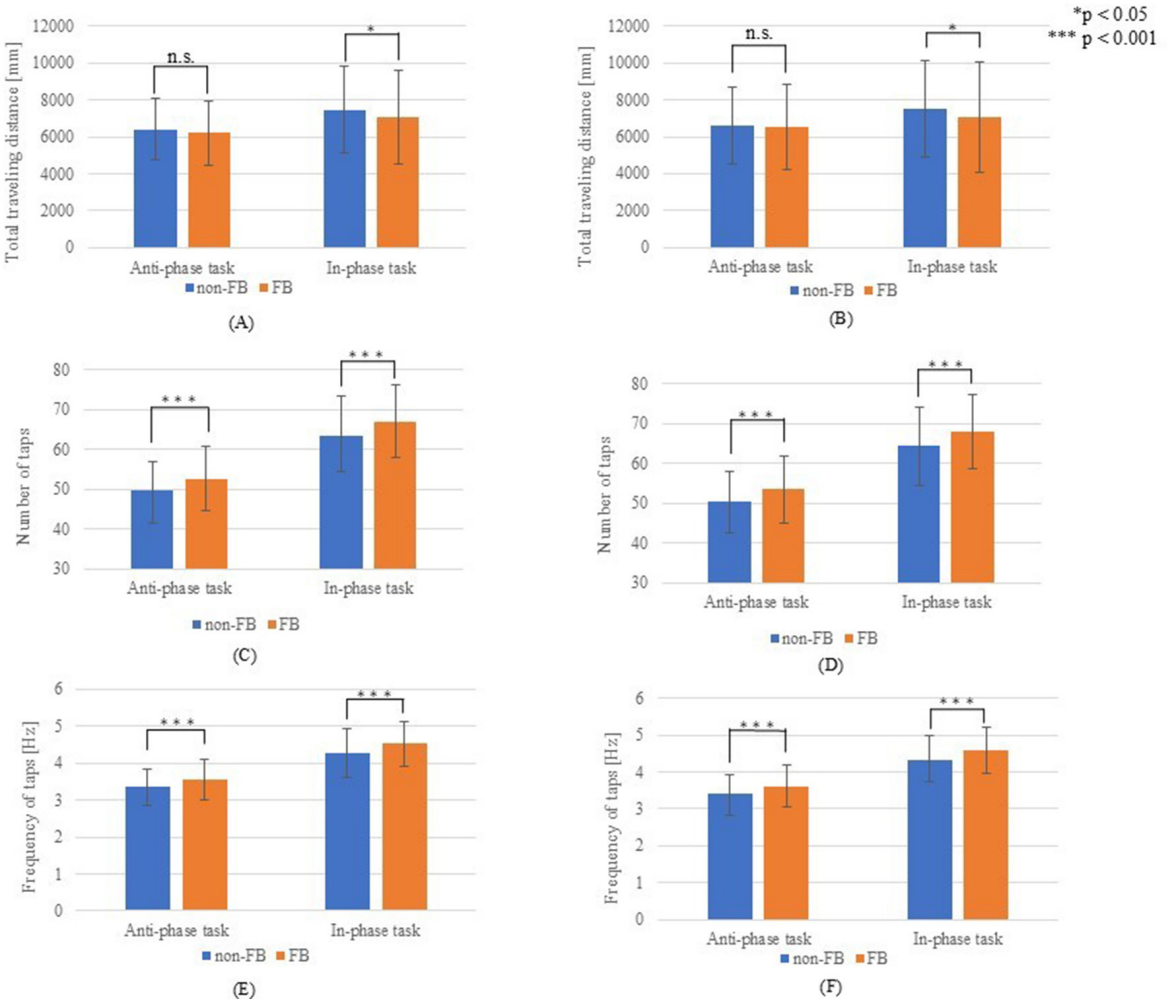
Results for hand movements. **(A)** Total travel distance of the non-dominant hand; **(B)** Total travel distance of the dominant hand; **(C)** Number of taps with the non-dominant hand; **(D)** Number of taps with the dominant hand; **(E)** Tap frequency of the non-dominant hand; **(F)** Tap frequency of the dominant hand.

**Figure 5:**
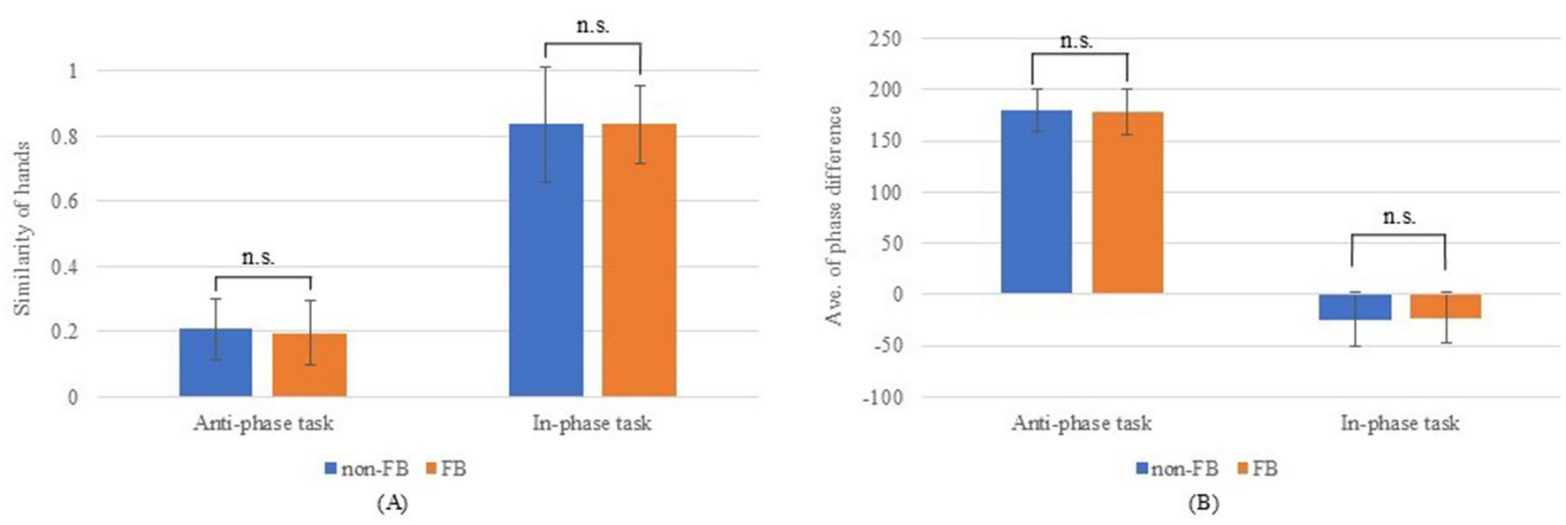
Results for left–right tapping balance. **(A)** Hand similarity; **(B)** Average phase difference.

Figure 4 compares the total distance traveled, number of taps, and tapping frequency with and without hand movement feedback. The total distance traveled was significantly greater without feedback for both simultaneous non-dominant and dominant movements (*p* < 0.05). Meanwhile, no significant differences were observed in alternating movements (non-dominant, *p* = 0.203; dominant, *p* = 0.554). By contrast, tapping frequency was significantly higher with hand movement feedback across all conditions (non-dominant and dominant, simultaneous, and alternating movements (*p* < 0.001). Figure 5 illustrates the comparison of the left–right balance in tapping, showing no significant differences in similarity of hands (anti-phase: *p* = 0.227; in-phase: *p* = 0.991) or average phase difference (anti-phase: *p* = 0.352; in-phase: *p* = 0.775).

Tables 4, 5 present the results of alternating and simultaneous opening and closing hand movements. The standard deviation of local maximum distance, an index of variation in hand opening and closing, was lower with feedback on the non-dominant hand during simultaneous movements. Variations in hand opening and closing were smaller on the dominant hand than on the non-dominant hand when feedback was present.

**Table 4:** Results of additional indicators in anti-phase.

	**Anti-phase task**	**Anti-phase task-FB**	***p* value**
SD of local max. distance (non-dominant hand)	9.4 ± 3.1 (8.3–10.5)	9.5 ± 2.9 (8.4–10.5)	-
Number of freezing calculated from acceleration (non-dominant hand)	23.1 ± 17.8 (16.7–29.5)	19.8 ± 15.6 (14.2–25.4)	-
SD of local max. distance (dominant hand)	7.9 ± 2.4 (7.0–8.8)	7.7 ± 2.5 (6.8–8.6)	-
Number of freezing calculated from acceleration (dominant hand)	18.2 ± 14.5 (13.0–23.5)	18.6 ± 17.2 (12.4–24.8)	-

“CV of local max. distance” is the value obtained by normalizing the variation of the amplitude by the mean value; the smaller the value, the better. The “Number of freezing calculated from acceleration (non-dominant hand)” expresses the freezing; the smaller the value, the better.

**Table 5:** Results of additional indicators in in-phase.

	**Anti-phase task**	**Anti-phase task-FB**	***p* value**
SD of local max. distance (non-dominant hand)	9.8 ± 4.4 (8.2–11.4)	9.2 ± 3.0 (8.1–10.3)	-
Number of freezing calculated from acceleration (non-dominant hand)	18.1 ± 15.1 (12.7–23.5)	14.8 ± 13.5 (9.9–19.6)	-
SD of local max. distance (dominant hand)	9.6 ± 7.0 (7.1–12.1)	6.9 ± 2.6 (5.9–7.8)	*p* < 0.05
Number of freezing calculated from acceleration (dominant hand)	18.1 ± 18.2 (11.5–24.6)	16.2 ± 15.1 (10.7–21.6)	-

SD, standard deviation.

## 5 Discussion

In finger tapping, both alternating and simultaneous movements were performed, with tapping performance being higher during simultaneous movements. Simultaneous exercises involve straightforward finger motions, whereas alternating exercises require separate movements on each side, making them more complex and challenging (Suzumura et al., 2021). Therefore, the results for alternating movements were lower, a finding consistent with previous studies (Sugioka et al., 2020).

In this study, finger movements are provided as waveform feedback. The horizontal axis represents time, allowing participants to obtain the speed of tapping, opening, and closing from the waveforms. In addition, participants could access hand and finger movement dynamics, such as speed and slowness. For left–right balance, participants who tap faster have smaller intervals between waveforms. Therefore, the left–right misalignment is difficult to confirm instantly. However, as feedback is provided for the left and right movements, the left and right movements can be checked independently of each other. In the present study, the number and frequency of tapping improved when feedback for hand movements was provided than when it was not provided. Visual feedback activates the motor cortex (Noble et al., 2013) and has been shown to improve motor performance (Shafer et al., 2019; Kim et al., 2021). The regions that control finger movements are considered to be the primary motor cortex, pre-motor cortex, and supplementary motor cortex (Sugioka et al., 2022). Therefore, finger tapping speed and number of taps were also improved in this study. Previous studies have similarly reported that feedback on movement-related information can improve performance, particularly in gait (Dobkin et al., 2010; Janakiraman et al., 2024). However, no significant difference in left–right balance was observed. This suggests that while hand movement feedback improved tapping speed, it did not affect left–right coordination. Visual feedback activates the motor cortex, but the pre-frontal cortex has been proven relevant for bimanual coordinated movements (Verstraelen et al., 2020). Therefore, balance may not have been improved. Alternatively, as healthy young adults, the participants in the present study possibly had good left–right balance without feedback and did not benefit from feedback. However, further validation is warranted.

Visual feedback during one-handed tapping movements improves tapping speed (Barallon et al., 2022). Additionally, real-time motion feedback for both hands improves overall hand function. For simultaneous movements, the total distance traveled was significantly lower with feedback, likely due to an increased number of taps resulting from the tapping width. However, no significant difference was found in the total distance for alternating movements, suggesting that feedback was more effective in this context. Regarding scooping movements, no improvement was observed in the dominant hand; however, a significant improvement was observed in the non-dominant hand, while the non-dominant hand showed significant gains. This improvement in the performance of the non-dominant hand may be attributed to the feedback helping to refine movement control. In addition, although the variations in finger opening and closing in the non-dominant hand did not improve, the dominant hand exhibited notable improvement. This suggests that the dominant hand, which generally has better control, benefited more from the feedback provided by the waveforms. Research indicates a correlation between finger tapping performance and brain volume, showing that lower tapping performance is associated with greater brain atrophy (Sugioka et al., 2022). Additionally, more complex hand movements and tasks requiring finger and manual dexterity activate brain function (Ota et al., 2020; Holper et al., 2009). A previous study focused on hand training in older adults and highlighted the importance of finger dexterity (Seol et al., 2023). In the present study, hand function was improved through motor feedback during alternating movements, suggesting that this technique can improve hand function and brain activity in older populations.

This study has three limitations. First, all participants were young adults. Although the effects of hand movement feedback were evident in this population, whether similar effects would be observed in older adults remains unclear. Moreover, individual differences are greater in older adults (Morse, 1993; Jiang et al., 2017). Thus, whether similar effects can be obtained must be tested. Second, the observed improvements in hand function were transient. The measurements taken during the study evaluated hand dexterity only while feedback was applied, leaving uncertainty regarding the permanence of these improvements. Motor learning is the process of learning a movement from practice and maintaining it for a long period. Yamamoto et al. (2022) suggested that simultaneous visual feedback is effective for motor learning in older adults. In this method as well, visual feedback is provided during finger tapping, which is expected to improve hand function. However, in previous studies of visual feedback, the duration of the experiment varied, such as only 1 day (Yamamoto et al., 2022) or multiple days with multiple times per day (Pak and Lee, 2020). Therefore, further validation, including the duration and number of experiments, is needed to verify the effectiveness and sustainability of the developed system. Third, the study relies solely on waveforms as feedback. In neurofeedback, alternative methods for presenting brain activity are available, such as mapping images (Barth et al., 2016), dynamic bar indicators (Mihara et al., 2012), and even virtual agents (Aranyi et al., 2016). Exploring these different presentation methods could lead to the discovery of more effective methods.

## 6 Conclusion

A system that provides feedback on finger movements during finger tapping tasks was constructed. Results confirmed that the system can implement feedback on hand movements and improve hand dexterity. Therefore, this system can contribute to the improvement of hand function. However, the present measurements were performed on healthy young participants. Thus, future research may need to verify the effectiveness of this system in older adults to determine its potential to improve hand function and investigate how cognitive function changes in intervention studies.

## Data Availability

The raw data supporting the conclusions of this article will be made available by the authors, without undue reservation.
